# Hedgehogs as a Potential Source of Zoonotic Pathogens—A Review and an Update of Knowledge

**DOI:** 10.3390/ani11061754

**Published:** 2021-06-11

**Authors:** Jakub J. Ruszkowski, Mateusz Hetman, Hanna Turlewicz-Podbielska, Małgorzata Pomorska-Mól

**Affiliations:** 1 Department of Animal Anatomy, Poznan University of Life Sciences, Wojska Polskiego 71C, 60-625 Poznań, Poland; jakub.ruszkowski@up.poznan.pl (J.J.R.); mateusz.hetman@up.poznan.pl (M.H.); 2 University Centre for Veterinary Medicine, Szydłowska 43, 60-656 Poznan, Poland; 3 Department of Preclinical Sciences and Infection Diseases, Poznan University of Life Sciences, Wołyńska 35, 60-637 Poznań, Poland; hanna.turlewicz@up.poznan.pl

**Keywords:** hedgehogs, zoonoses, infections

## Abstract

**Simple Summary:**

Up to date studies indicate that wild hedgehogs may act as carriers and/or hosts for bacterial, viral, and fungal pathogens with zoonotic potential, posing a significant threat to humans. The same applies to domestic hedgehogs, which are increasingly kept as pets. Considering the potential risk of infection to humans through close contact with hedgehogs or the contaminated environment they inhabit, current data on the prevalence of various zoonotic pathogens in these animals is desirable.

**Abstract:**

Hedgehogs are small insectivorous mammals common across Europe, Asia, and Africa. The increased encroachment of humans into hedgehog habitats has disrupted the human-animal-environment interface. With growing interest in the zoonotic diseases of wildlife species, more studies have been devoted to this subject in the last few years. These papers provide information about known and new emerging diseases. Here we review the current knowledge regarding bacterial, viral, protozoic, and mycotic pathogens with zoonotic potential and assess the importance of hedgehogs as their carriers. Both wild and pet hedgehogs were included in the review. Data from several countries and various hedgehog species were included. The study shows the importance of hedgehogs as carriers of zoonotic diseases and reservoirs of zoonotic pathogens in varied habitats.

## 1. Introduction

Hedgehogs are small and nocturnal insectivorous mammals spread widely across Europe, Asia, and Africa [1]. To date, 16 species of hedgehogs are known, placed into five genera: *Hemiechinus* (two species), *Atelerix* (four species), *Erinaceus* (four species), *Paraechinus* (four species), and *Mesechinus* (two species) [2].

*Erinaceus* and *Atelerix* are the most commonly described genera. European and African hedgehogs are more often kept as companion animals. Since European hedgehogs are protected by law in many European counties, which have signed the Berne Convention on the Conservation of European Wildlife and Natural Habitats, keeping them as pets without special permissions is illegal in these countries [1]. Wild animals, including hedgehogs, often become accidental victims of human activity, and their carcasses can serve as a valuable source of data for epidemiological studies focused on zoonoses in urban environments [3]. Wild hedgehogs have become frequenters in cities, where they search for food and shelter [4]. Living in parks, home gardens, and other urban green areas leads to exposure to different anthropogenic endangerments, i.e., wildlife-vehicle collisions, intoxication with chemicals used in urban green areas after ingesting poisoned snails (i.e., molluscicides), and cats and dogs, or other predator attacks [5,6]. Hedgehogs in cities are active both at night and during the day. People take hedgehogs home or to wildlife rehabilitation centers in order to rescue them, exposing themselves to potential contact with various zoonotic pathogens that hedgehogs are carriers of (Table 1). Hedgehogs also contaminate the grass environment with their feces, in which various pathogens (including *Salmonella* spp) may be present [7]. Subsequently, a human infection may occur (directly from pathogen-contaminated grass) or indirectly through various mechanical vectors (e.g., dogs and cats) [7,8].

This paper is a literature review of pathogens with zoonotic potential in domestic and wild hedgehogs (Table 2), which is important from scientific and practical points of view, especially for employees of wildlife rehabilitation centers, people involved in the rescue of wild hedgehogs, or for owners of domestic hedgehogs. For some pathogens described in this review, no research has yet been published confirming their transmission from hedgehogs to humans. However, given that they are pathogenic for humans and that their presence in hedgehogs has been confirmed, this possibility cannot be excluded.

## 2. Bacterial Zoonotic Pathogens

### 2.1. Corynebacterium *spp.*

To date, only one report of isolation of an unidentified *Corynebacterium* spp. in a domestic African pygmy hedgehog in the US has been published [12]. This pathogen was identified in lung tissue collected from the dead animal at necropsy. *Corynebacterium pneumonia* was considered the cause of the hedgehog’s death [12]. Taking into account the pathogenicity of this bacteria in humans, the possibility of transmission of the pathogen from hedgehogs to humans is likely, and therefore it requires further investigations. 

### 2.2. Coxiella Burnetii

*Coxiella burnetii* is an obligate, intracellular, Gram-negative bacterium causing the Q Fever zoonotic disease [36]. Livestock and wild animals are considered as a reservoir and/or carriers of the pathogen. Infection in humans occurs mainly through direct contact with infected animals or their excretions [48], although other vectors, including ticks, have also been suggested to transmit the disease. Contaminated urine, feces, milk, and postpartum fomites are potential sources of infection in humans.

Gong et al. [36] investigated the prevalence of *C. burnetii* in amur hedgehogs (*Erinaceus amurensis*) from Hubei province. Forty-one individuals were euthanized, and samples of heart, liver, spleen, kidney, and lung were collected. Samples were tested for *C. burnetii* with PCR targeting three genes (com1, rrs, and icd), followed by multispacer sequence typing (MST), and 12.2% (5/41) hedgehogs were PCR positive for *C. burnetii*. Sequencing revealed the presence of two novel genotypes of *C. burnetii*. Phylogenetic analysis revealed the strains were similar to a group of isolates from Q fever chronic patients and other mammals [36]. The study showed that *C. burnetii* were highly prevalent in hedgehogs, suggesting that hedgehogs may also play an important role in the epidemiology and transmission of *C. burnetii* to humans. In traditional Chinese medicine, products from hedgehogs are used in treatments for hemorrhoids [36]. In addition, these animals are eaten in some regions (i.e., central China). Gong et al. [36] suggest that humans may be infected with *C. burnetii* when they come into contact with hedgehogs, such as via inhaling the contaminative aerosol in the process of capturing or slaughtering [36].

### 2.3. Leptospira *spp.*

*Leptospira* is one of the two genera in the family Leptospiraceae. These genera comprise etiological agents of leptospirosis, a zoonotic disease of increasing importance [49]. Different species of *Leptospira* spp. were isolated from wild hedgehogs in Europe, Asia, and New Zealand [9,25,37].

In a study by Ayral et al. [25], kidney samples from 112 wild hedgehogs from France were subjected to PCR analysis; 42/112 (37.5%) samples were positive for *Leptospira* spp. and this was the highest prevalence of all 28 tested animal species, while 41 individuals carried *Leptospira interrogans* and one carried *Leptospira borgpetersenii*. Ma et al. [37] examined kidney samples collected from 41 wild Amur hedgehogs (*Erinaceus amurensis*) from China. Results confirmed the presence of an *L. interrogans* gene in 19.5% (8/41) of samples and three genes tested in 7.5% (3/41) of samples [37]. In the study performed by Brockie and Till [9], kidney samples collected from 78 hedgehogs living near dairy cattle farms were subjected to microbiological analyses, and *Leptospira ballum* was isolated from 5/78 (6%) of the kidney samples.

Given that bacteria from the Leptospiraceae family have been repeatedly isolated from hedgehogs (their detection in kidneys may lead to probable excretion with urine), this area and the zoonotic potential of hedgehogs in this regard requires further research. It cannot be excluded that they play a role in the circulation of *Leptospiraceae* spp. in wild animals (including rodents), which can infect humans.

### 2.4. Mycobacterium *spp.*

Hedgehogs were also identified as carriers of *Mycobacterium* spp. *Mycobacteria* are Gram-positive, acid-fast bacteria, causing several infectious diseases in humans and animals [50].

*Mycobacterium avium subs. paratuberculosis* is a causative agent of paratuberculosis in ruminants (Johne’s disease) [51]. The link between infection with this pathogen and human disease has been theorized for many years, with Crohn’s disease being one of many suspected resultant conditions [52,53]. *Mycobacterium avium subs*. *paratuberculosis* was found in 15/42 (36%) wild hedgehogs caught on three deer farms affected with Johne’s disease in New Zealand [26]. Infection with *Mycobacterium avium subs*. *paratuberculosis* in the gastrointestinal tract was confirmed in hedgehogs. Gross pathological changes were identified in the gastrointestinal tract of six hedgehogs [21]. This study did not determine whether these animals were able to shed the pathogen into the environment; however, this cannot be ruled out.

The results of another study conducted in New Zealand, an area endemic for bovine tuberculosis, showed that wild hedgehogs might play a significant role in the epidemiological chain of this disease [27]. In the study mentioned, 79 hedgehogs were dissected in order to analyze macroscopic lesions. Tissue samples taken from sixteen selected hedgehogs were subjected to bacteriological tests. *Mycobacterium bovis* was isolated from 4/79 (5.1%) hedgehogs and *M*. *avium* in 1/79 (1.3%), respectively. Hedgehogs, not only as prey species but also as scavengers, can become infected with *Mycobacterium* spp. in a variety of ways, which makes them an important element in the epidemiology of the infections caused by these bacteria [27]. To date, two reports confirming *Mycobacterium marinum* in hedgehogs have been published [13,54]. *Mycobacterium marinum* was isolated from Japan from a domestic African pygmy hedgehog (*Atelerix albiventris*) with eosinophilic leukemia [13] and from a European hedgehog kept as a pet [54].

### 2.5. Methicillin-Resistant Staphylococcus aureus

Another important zoonotic pathogen, isolated from hedgehog carriers, is methicillin-resistant *Staphylococcus aureus* (MRSA) [8,15,29]. MRSA, resistant to most β-lactams, is a major concern due to causing hospital-associated infections in humans worldwide [55,56]. Additionally, livestock-associated MRSA (LA-MRSA) is an emerging problem worldwide and was recognized as one of the most important causes of MRSA infections in humans [55,57,58]. MRSA, encoding the mecC gene, is an important pathogen for human medicine since it may be misdiagnosed as methicillin-sensitive *S. aureus*, with important potential consequences for individual patients and MRSA surveillance [56]. Different MRSA strains were isolated from wild hedgehogs in Europe [8,15,29].

A study performed in New Zealand showed an 85% prevalence of *S. aureus* in hedgehogs, and a high rate of penicillin resistance strains (86.3%) was detected [46]. In 2003, MRSA was isolated from hedgehogs in the United Kingdom; however, no typing data on this isolate were available [59].

Monecke et al. [8] examined two hedgehogs in Sweden (one found dead and one euthanized). Samples of brain and kidney from one and samples of skin from the other animal were cultured with routine bacteriological methods, and PCR and sequencing were also performed [8]. ST130-MRSA-XI isolates were found from samples from both hedgehogs. The MRSA infection described in the mentioned study caused severe disease in the hedgehogs. One of the hedgehogs had developed lethal septicemia, and MRSA was isolated from abundant growth in pure culture from all the samples analyzed. The second hedgehog developed severe dermatitis [8]. In both cases, given the symptoms observed in the animals and the severity of the infection, shedding of the bacteria and contamination of the environment by MRSA appear to be feasible.

The isolates were identical to previously described isolates from humans [8,60]. The recent findings suggest that CC130 might be a zoonotic lineage of *S. aureus* and that SCCmec XI/mecC may be attributed to zoonotic origin [8].

In a study by Bengtsson et al. [15] conducted in Sweden, with the use of nasal, oral, and perineal swabs, MRSA was isolated from 35/55 (64%) hedgehogs residing at wildlife rescue centers. All MRSA isolates found in the study mentioned carried the mecC gene.

The aim of the study by Rasmussen et al. [29] was to determine the MRSA prevalence in European hedgehogs in Denmark as well as to investigate the determinants of MRSA carriage in hedgehogs and to determine the potential of MRSA zoonotic transmission from hedgehogs to humans. In addition, MRSA isolates obtained from hedgehogs were characterized at both phenotypic and molecular levels. For this purpose, 188 nasal swabs were taken from dead hedgehogs collected throughout Denmark, and 114/188 (61%) of the animals carried mecC-MRSA, whereas none carried mecA-MRSA. Two genetic lineages of mecC-MRSA were determined—CC130 and CC1943. The zoonotic transmission of MRSA was not confirmed in the above study, although more than half of hedgehogs were MRSA carriers, which makes this species an important marker of the MRSA prevalence in wildlife [29].

### 2.6. Salmonella *spp.*

*Salmonella* spp. are facultative, anaerobic, Gram-negative, rod-shaped bacteria belonging to the family Enterobacteriaceae [61]. Salmonellosis is the most often described zoonotic bacterial disease in hedgehogs. *Salmonella enterica* serovar Enteritidis (*S.* Enteritidis) is one of the most common types of *Salmonella* that cause infections in humans and animals [47].

*Salmonella* was detected in samples from both pet and wild hedgehogs [47,62,63]. *S.* Enteritidis and *S.* Typhimurium were the most prevalent serovars found in hedgehogs [28,47]. Hedgehogs infected with this pathogen can display various clinical symptoms, i.e., anorexia, weight loss, and diarrhea, though some may remain asymptomatic carriers [38]. To date, numerous cases of salmonellosis in humans have been described, possibly linked to both pet and wild hedgehogs [7,38,41,47,63]. Human salmonellosis following direct contact with hedgehogs was described in Canada [62], United States [10,63], Great Britain [8], Norway [38,39], and Japan [41]. According to Kagambèga et al. [7], hedgehogs can serve as reservoirs of *Salmonella* spp. in various ways. In some African countries, where hedgehogs are a source of food, meat from infected hedgehogs is one of the potential routes of infection for people [7]. Wild hedgehog feces can contaminate water sources (rivers and wells), especially during the rainy season in an equatorial climate. In several areas, such water is consumed without any treatment and thus exposes people to various pathogens. However, cases of human infection from hedgehogs were also described in highly developed countries where hedgehogs are kept as pets or treated in wildlife centers [64]. In these cases, the most commonly identified *Salmonella* serotype was *S.* Tilene [40], and this serotype was also found in cattle and chickens [7,65]. The cohabitation of these animals in the same environment (e.g., pasture) poses a high risk of transmission of different pathogens between wildlife and livestock, increasing the risk of entering the pathogens into the food chain. A study performed by Kagambèga et al. [7] in Burkina Faso confirmed that hedgehogs are also carriers of numerous *Salmonella* serotypes found in farm animals [7]. Monitoring of wild mammals (including hedgehogs) health status, especially around farms affected with salmonellosis, may be important for the more detailed understanding of the disease epidemiology and identification of new potential reservoirs. Compliance with the principles of strict biosecurity rules and basic hygiene (washing hands) after any contact with wild animals, including hedgehogs, is recommended to reduce the risk of *Salmonella* spp. transmission from hedgehogs to humans [10].

Handeland et al. [38] investigated the fecal carriage of *Salmonella* in Norway. In the study mentioned, 320 wild hedgehogs were caught during the night, placed into boxes for about 2 h, and fed. The study took place over four sampling sites. The samples collected in two out of four sites were negative, while in the next two sites, 39% and 41% of the hedgehogs were infected with a monophasic variant of either serovar Typhimurium (4,5,12:i:1,2) respectively, one year after a human outbreak of salmonellosis or during the outbreak. Both outbreaks in people were caused by a monophasic variant of either serovar Typhimurium (4,5,12:i:1,2). Prevalence of the pathogen was higher (71%) in animals sampled near garden feeding stations than elsewhere (gardens, parks, road edges, and groves) (25%).

Lawson et al. [47] investigated various tissue samples (liver and small intestinal contents) from 170 wild hedgehogs and fecal samples from 208 wild hedgehogs in Great Britain. Classical microbiological methods and Gram staining coupled with the determination of biochemical characteristics and histopathological examination were used to identify bacterial isolates and description of lesions. *S.* Enteritidis multi-locus sequence-type (ST)183 was isolated from 46/170 (27%) tissue samples and from 6/208 (3%) fecal samples. The types of *Salmonella* found in hedgehogs were also investigated and compared with those found in humans. The results showed that infections in both species might have originated from a common population. These results confirmed that hedgehogs might serve as a reservoir host and source of salmonellas for humans [47].

The role of hedgehogs as carriers of *Salmonella* spp. has also been documented in domestic hedgehogs [66]. Anderson et al. [66] described multi-state outbreaks of human salmonellosis in the United States between 2011 and 2013, for which hedgehogs kept as companion animals were the most likely source of the pathogens. Analyses showed that approximately 80% of patients included in the study reported contact with a pet hedgehog in the week prior to disease onset. A thorough epidemiological investigation identified sources of hedgehog purchase; however, in the end, a specific source of infection could not be identified [66].

### 2.7. Streptococcus *spp.*

*Streptococcus pyogenes (S. pyogenes)* is a Gram-positive, facultative, and anaerobic bacteria [30]. To date, one case of infection of a wild hedgehog by *S. pyogenes* has been published [30]. A dead European hedgehog was found in a suburban garden in England with severe dental lesions and abscesses in several tissues. *S. pyogenes* was isolated from the right deep cervical lymph node, hepatic abscess, peritoneal cavity, and pleural cavity. The infection with this pathogen was considered to be the cause of the hedgehog’s death. The authors concluded that the dental lesions might have been the site of entry of the *S. pyogenes* into the blood, with which it spread to other tissues. Further analysis confirmed that *S. pyogenes* emm 28, a strain with zoonotic potential, was responsible for the infection in the case described. It was the first report of *S. pyogenes* infection in a free-living hedgehog and the first report of the emm 28 strain in a non-human species [30]. The authors of the above-mentioned study speculated that infection in the described case occurred due to anthroponotic transmission; however, there is no clear evidence for this statement.

Rodenbaugh et al. [16] described *Streptococcus* infection in an African pygmy hedgehog with skin lesions. In this case, the *Streptococcus dysgalactiae* was identified, belonging to Lancefield Group A [16], and *S. dysgalactiae* is known to be pathogenic for humans [67].

### 2.8. The Hedgehog as a Carrier of Bacterial Pathogens Transmitted by Ticks

Due to the high tick infestation in wild hedgehogs, these animals are highly exposed to various tick-borne pathogens that may have zoonotic potential, not to mention the host‘s health. Several studies demonstrated that hedgehogs serve as animal hosts for many ticks, including *Ixodes ricinus* and *Ixodes hexagonus* [22,23]. These tick species are able to transmit various pathogens, including zoonotic ones such as *Borrelia* spp., *Rickettsia helvetica*, and *Anaplasma phagocytophilum* [22]. The prevalence of various pathogens differs geographically; however, in most research *A. phagocythophilum* and *Borrelia* spp. are the most common zoonotic tick-borne pathogens found in ticks collected from the hedgehogs. The role of hedgehogs in the epidemiology of infections with tick-borne pathogens is not fully recognized, nevertheless given the increasing number of hedgehogs (and thus ticks) in the human environment; further studies focused on this area are certainly required. In particular, an assessment of the involvement, degree, and extent of hedgehog contribution to the enzootic cycle of these pathogens is needed, as well as a description of their transmission mechanisms and cycles involving hedgehogs and ticks.

#### 2.8.1. *Anaplasma phagocythophilum*

*Anaplasma phagocythophilum* is an important, zoonotic, tick-borne bacteria causing granulocytic anaplasmosis in humans, ruminants, horses, and dogs [22]. There are different strains of *A. phagocythophilum* circulating among wildlife and domestic animals with different host tropisms and pathogenicity [68]. The main vector of *A. phagocythophilum* in Europe is *I. ricinus*. Hedgehogs act as propagation hosts for *I. ricinus*; hence, these animals might be responsible for tick maintenance in urban areas and the epidemiology of anaplasmosis [69].

In the study by Lesiczka et al. [22], ear tissue, muscle, lungs, liver, spleen, urinary bladder, kidney, brain, and blood samples were collected from hedgehog carcasses. Blood and skin samples were also collected from live-trapped hedgehogs. Samples were tested for the presence of *A. phagocytophilum* DNA by quantitative PCR (qPCR). The result was considered positive when at least one tissue sample was positive in qPCR. The prevalence of *A. phagocytophilum* was high in both *E. europaeus* and *E. roumanicus*. In *E. europaeus* species, it reached 97.6% of carcasses and 93.1% of living animals, whereas in E. *roumanicus* it reached 97.6% and 85.7%, respectively [22].

Jahfari et al. [33] investigated the prevalence of various tick-borne pathogens, including *A. phagocythophilum*, in ticks collected from hedgehogs in Belgium. Prevalence of the different pathogens in ticks, including engorged and questing ticks, were also investigated. The presence of *I. hexagonus* and *I. ricinus*, at all life stages was confirmed on hedgehogs included in the study. The presence of genetic material of A. *phagocytophilum* and *R. helvetica* was confirmed in both *I. hexagonus* and *I. ricinus*. *A. phagocytophilum* was more frequently detected in engorged than in questing *I. ricinus*.

In the study by Jahfari et al. [33], over 71% of ticks were infected with at least one of the pathogens studied. Of these infected ticks, the presence of only one pathogen was confirmed in 61% of ticks, while in the remaining infected ticks, the genetic material of at least two pathogens was detected. *A. phagocytophilum* and *R. helvetica* were found in 38.7% of all analyzed ticks from 34 hedgehogs and in 40% of all analyzed ticks from 37 hedgehogs, respectively. Infection with *Borrelia burgdorferi* (s.l.) was confirmed in 24.7% of all analyzed ticks from 28 hedgehogs. Infection with at least one investigated pathogen was detected more frequently in *I. ricinus* (81.9% of the tested ticks) than in *I. hexagonus* (70.7% of the tested ticks). There were no differences in the prevalence between adult and juvenile hedgehogs for any of the studied pathogens [33].

In a study conducted in the Netherlands by Krawczyk et al. [34], *A. phagocythophilum* was found in 74/277 (27%) *I. hexagonus* and 6/25 (24%) *I. ricinus* collected from living hedgehogs [34].

#### 2.8.2. *Borrelia* spp.

The *Borrelia burgdorferi* s.l. complex consists of at least 12 species, three of which are assumed to be pathogenic to humans and are causative agents of Lyme borreliosis—the most common tick-borne disease in Europe and the US [70]. Another important zoonotic pathogen is *Borrelia miyamotoi*—a causative agent of human nonspecific febrile illness [71].

Jahfari et al. [33] revealed the presence of genetic material of *Borrelia miyamotoi* and species of *Borrelia burgdorferi* (*B. afzelii, B.bavariensis*, and *B. spielmanii*) in both *I. hexagonus* and *I. ricinus* sampled from hedgehogs. *B. afzelii*, *B. bavariensis*, and *B. spielmanii* were more frequently detected in engorged than in questing *I. ricinus*.

The study conducted by Skuballa et al. [35], with the use of samples from 269 dead wild hedgehogs from different locations in Europe and Great Britain and three hedgehogs from an experimental population, showed 13.8% (37/269) prevalence of *Borrelia burgdorferi* s.l. complex in a European population of hedgehogs. Majerová et al. [24] described that 5% of investigated hedgehog tissues were positive for DNA of *B.miyamotoi* [24].

The results obtained to date indicate that hedgehogs may contribute to the spread and transmission of tick-borne pathogens in urban areas. The relatively high prevalence of *Borrelia* spp., *A. phagocytophilum* in ticks collected from hedgehogs suggests that hedgehog‘s involvement in the endemic occurrence of these pathogens in urban areas might be significant. However, the question of the hedgehog‘s role in the self-sustaining and circulation of the above-mentioned pathogens remains open.

## 3. Viral Zoonotic Pathogens

### 3.1. Severe Fever with Thrombocytopenia Syndrome Virus (SFTSV)

SFTSV is an etiologic agent of severe fever with thrombocytopenia syndrome (SFTS), an emerging hemorrhagic fever affecting humans and cats [72,73]. SFTSV belongs to the genus *Banyangvirus*, family *Phenuiviridae*, order *Bunyavirales* [42,74]. SFTS was first reported in 2009 in China [75] and later in Japan [76] and Korea [77]. Since then, new cases have been reported in Vietnam and Taiwan [78,79]. SFTS is mainly transmitted by ticks, which infest a variety of animals [80,81,82]. Viral RNA and/or specific antibodies against SFTSV have been detected in various species, including wild and domestic animals [60].

In a study by Sun et al. [82], sera collected from 14 hedgehogs were tested for SFTSV antibodies using ELISA, and 64.3% of serum samples were positive for SFTSV IgG. No viral RNA was detected in any of the samples. These results suggest that hedgehogs may serve as a potential animal host of the SFTSV, albeit may not be permanently infected [82].

### 3.2. Tick-Borne Encephalitis Virus

Tick-borne encephalitis virus (TBEV) is an etiological agent of tick-borne encephalitis (TBE), a potentially fatal neurological infection affecting humans in Europe and Asia in cycles involving ticks and wild vertebrate hosts [83]. Tick-borne encephalitis virus was confirmed for the first time in the northern white-breasted hedgehog (*Erinaceus roumanicus*) by Kozuch et al. [43]. TBEVs were identified in blood collected from live-trapped animals [43]. In another study [84], TBEV was shown to able to persist in hibernating hedgehogs, which indicates hedgehogs could act as important long-term reservoirs for the pathogens mentioned [84].

In the study performed by Schönbächler et al. [44], various organs, blood, and ticks collected from 65 European hedgehogs were analyzed for the presence of TBEV. Lung, liver, spleen, and kidney samples from 56 hedgehogs and 114 infesting ticks were used for the detection of viral RNA. In addition, 19 blood samples were tested for specific antibodies by ELISA. Antibodies specific to TBEV were detected in one hedgehog with neurological symptoms. Low levels of viral RNA were detected in the lung and spleen in the same animal in RT-PCR. No viral RNA was detected in any of the ticks. The authors concluded that the simultaneous detection of antibodies, viral RNA, and clinical signs in the same animal indicates that the European hedgehog is probably a competent host for TBEV and allows the assumption that this species may act as a reservoir [44].

Considering the occurrence of TBEV in hedgehogs, there is a potential risk for its transmission by ticks from hedgehogs to humans.

### 3.3. Other Viruses

More recently, a wide variety of coronaviruses [85,86,87] and Belerina viruses [88] have been found in hedgehogs in many countries, and their zoonotic capacity has not yet been confirmed. However, given their belonging to *Coronaviridae* or *Paramyxoviridae* family, their zoonotic potential should be analyzed.

## 4. Zoonotic Fungal Agents

### 4.1. Candida Albicans

Another significant mycotic agent that affects hedgehogs is *Candida albicans*, recorded as a commensal of the digestive tract [89]. Candidiasis is a well-known human disease and, according to presented data, hedgehogs should also be considered as potential carriers or reservoir hosts of *Candida albicans*. Reports of oral and intestinal candidiasis in *Erinaceus europaeus* [32] and Pygmy hedgehogs [20] have been published. Moreover, English et al. [21] described footpad invasion by *Candida albicans* in *Erinaceus albiventris*. These results indicate the potential of hedgehogs as infection carriers and indicate that public health institutions should not neglect this important source of zoonotic transmission of *Candida albicans* to humans.

### 4.2. Microsporum *spp.*

*Microsporum* is a dermatophyte genus regarded as a significant zoonosis. *Microsporum* spp., in particular, *M. canis* and *M. gypseum*, are common agents with a worldwide spread that cause dermatophytosis in humans [90]. To date, Iacob and Iftinca [17] have described the only published case of African pygmy hedgehog acarosis induced by *Caparinia tripilis* with accompanying *Microsporum* spp. infection cultured from lesioned cutaneous tissues.

### 4.3. Trihophyton erinacei

*Trichophyton erinacei (T. erinacei*), previously described as *Trichophyton mentagrophytes* var. *Erinacei*, is the major mycotic agent transmitted by hedgehogs. First mentioned in 1960 [73], it was isolated from human skin infection. There are several reports describing *T. erinacei* infections in humans [18,45,91,92,93,94]. A factor that may contribute to the transmission of fungal infection from hedgehogs to humans is a body covered with spines. Thus, handling hedgehogs without gloves greatly increases the risk of dermatophytosis in humans. Numerous patients in the listed reports mentioned contact with pet [45,91,92] or wild hedgehogs [18,93,94]. There are also papers confirming the ability of the hedgehog to transmit dermatophytes to humans [18,95]. European hedgehogs, as well as Four toed-hedgehogs and other hedgehog species, are carriers of *T. erinacei*. Clinical signs characteristic for *T. erinacei* infection in wild European hedgehogs are described as crusty lesions, alopecia, and loss of spines, mainly on the head area, although the course of infection may be mild or asymptomatic [96]. Smith and Marples [46] examined 114 European hedgehogs in New Zealand suburbs. Materials from hair and spines were cultured on Sabouraud’s agar plates. Growth of *T. erinacei* strains was observed after culture of 44.7% (51/114) samples. Smith and Marples [46] found that *Caparinia ripilis* was responsible for the scabbiness of most hedgehogs, and that *T. erinacei* was usually recoverable from such lesions. In addition, mites were considered capable of transmitting ringworm from one animal to another. However, most new infections were attributed to contact between infected mother and offspring. According to English [11], the prevalence of *T. erinacei* infection in British hedgehogs was approximately 30%.

In the research conducted in Spain [19], 20 pet hedgehogs suspected of suffering from dermatophytosis were examined. Microbiological and molecular examination revealed that 50% of the animals were *T. erinacei* positive.

## 5. Conclusions

The occurrence of many pathogens in hedgehogs, including zoonotic ones, and the existence of hedgehogs in close proximity to humans, means that this species should be considered as an important part of the epidemiology of various zoonotic infections. Hedgehogs often inhabit suburban gardens and parks, being closer to humans. Given the common close contact with hedgehogs, current data on the prevalence of various zoonotic pathogens in these animals is important and desirable. Assessing the potential risk of infection to humans through contact with hedgehogs or the contaminated environment they inhabit also requires further research.

## Figures and Tables

**Table 1:** Zoonotic pathogens isolated from clinically healthy, sick, and dead hedgehogs or animals with no clinical data.

Clinically Healthy *	Sick **	Dead	No Clinical Data
*Leptospira ballum* [9] *Salmonella enterica* [7] *Salmonella tilene* [10] *Trichophyton erinacei* [11]	*Corynebacterium sp* [12] *Mycobacterium marinum* [13] *Staphylococcus aureus* [14] *Staphylococcus aureus (MRSA)* [8,15] *Streptococcus dysgalactiae* [16] *Microsporum* spp. [17] *Trichophyton erinacei* [18,19] *Candida albicans* [20,21]	*Anaplasma phagocythophilum* [3,22] *Borrelia burgdorferi sensu lato* [3,23] *Borrelia miyamotoi* [24] *Leptospira interrogans* [25] *Leptospira borgpetersenii* [25] *Mycobacterium avium* subs. *paratuberculosis* [26] *Mycobacterium bovis* [27] *Rickettsia helvetica* [3] *Salmonella* Typhimurium [28] *Staphylococcus aureus (MRSA)* [8,29] *Streptococcus pyogenes* [30] *Trichophyton erinacei* [28,31] *Candida albicans* [32]	*Anaplasma phagocythophilum* [33,34] *Borrelia burgdorferi* sensu lato [33,34,35] *Coxiella burnetii* [36] *Leptospira interrogans* [37] *Rickettsia helvetica* [33] *Salmonella* typhimurium [38,39] *Salmonella enterica* [34] *Salmonella tilene* [40] *Salmonella* Stanley [41] SFTSV [42] TBEV [43,44] *Trichophyton erinacei* [31,45,46]

* Clinically healthy: animals with no clinical signs of disease, ** Sick: animals displaying clinical signs of infection.

**Table 2:** Zoonotic pathogens isolated from wild and pet hedgehogs.

	Bacterial	Viral	Fungal
Wild hedgehogs	*Anaplasma phagocytophilum* [3,22,33] *Borrelia burgdorferi sensu lato* [3,22,33,34] *Borrelia miyamotoi* [24] *Coxiella burnetii* [36] *Rickettsia helvetica* [3,33] *Leptospira interrogans* [25,37] *Leptospira ballum* [9] *Leptospira borgpetersenii* [25] *Mycobacterium avium* ssp. *paratuberculosis* [26] *Mycobacterium bovis* [27] *Staphylococcus aureus* [14] *Staphylococcus aureus (MRSA)* [8,15,29] *Salmonella* Enteritidis [47] *Salmonella* Typhimurium [28,38,39] *Streptococcus pyogenes* [30]	TBEV [43,44] SFTSV [42]	*Candida albicans* [32] *Trihophyton erinacei* [11,28,31,46]
Pet hedgehogs	*Corynebacterium* sp. [12] *Mycobacterium marinum* [13] *Salmonella* Stanley [41] *Salmonella tilene* [10,40] *Streptococcus dysgalactiae* [16]		*Candida albicans* [20,21] *Microsporum* spp. [17] *Trihophyton erinacei* [18,19,45]

## Data Availability

Not applicable.
